# The mechanism of impaired delayed recall verbal memory function in Parkinson's disease with orthostatic hypotension: a multiple imaging study

**DOI:** 10.3389/fneur.2023.1149577

**Published:** 2023-07-18

**Authors:** Xiaofan Xue, Anqi Huang, Jingrong Zeng, Haixia Song, Yingqi Xing, Piu Chan, Erhe Xu, Lichun Zhou

**Affiliations:** ^1^Department of Neurology, Beijing Chaoyang Hospital, Capital Medical University, Beijing, China; ^2^Department of Neurology, Beijing Xuanwu Hospital, Capital Medical University, Beijing, China; ^3^Department of Neurology, The People's Hospital of Shijiazhuang, Shijiazhuang, Hebei, China

**Keywords:** Parkinson's disease, orthostatic hypotension, cognitive impairment, imaging technique, memory function

## Abstract

**Introduction:**

Orthostatic hypotension (OH) frequently accompanies autonomic dysfunction and is an important risk factor for cognitive impairment in Parkinson's disease (PD). However, the association between different cognitive functions and OH in PD patients is not yet fully understood.

**Methods:**

This study aimed to evaluate the scores of different cognitive domains and multiple parameters using different imaging techniques on PD patients with or without OH. A total number of 31 PD patients with OH (*n* = 20) and without OH (*n* = 11) were recruited from the Department of Neurology, Beijing Xuanwu Hospital for this study. All patients underwent beat-to-beat non-invasive blood pressure recordings and an active standing test to evaluate neurogenic OH and a global neuropsychological test to assess cognitive function. All patients underwent dynamic cerebral autoregulation (dCA) measurement, brain magnetic resonance imaging (MRI), and brain 18fluorine-fluorodeoxyglucose positron emission tomography/computed tomography (18F-FDG PET/CT).

**Results:**

The results showed that OH patients had poor delayed recall verbal memory when compared with the PD patients without OH (1.75 ± 1.59 vs. 3.10 ± 1.73, *p* = 0.042). The dCA test indicated a significant difference in the right very low-frequency (VLF) gain between two groups (1.27 ± 0.17 vs. 1.10 ± 0.26, *p* = 0.045) and the brain 18F-FDG PET/CT indicated a significant difference in the SUV (right medial temporal lobe) to SUV (occipital lobe) ratio (0.60 ± 0.08 vs. 0.67 ± 0.11, *p* = 0.049). Meanwhile, these two imaging parameters were negatively correlated (*p* < 0.001). Furthermore, the score of a delayed recall verbal memory in the OH group was positively correlated with the right medial temporal lobe to occipital lobe ratio (*p* < 0.001) and was negatively correlated with the right VLF gain (*p* = 0.023).

**Discussion:**

PD with OH patients had poor delayed recall memory, which might have been caused by the decreased metabolic dysfunction of specific medial temporal lobe due to the impaired dCA ability.

## 1. Introduction

Parkinson's disease (PD) is commonly accepted to be associated with various non-motor symptoms, including sleep disturbance, cognitive impairment, and autonomic dysfunction (1). Autonomic impairment associated with PD is characterized by clinical features of constipation, sweating, orthostatic hypotension (OH), and postprandial hypotension (PPH), even in the early phase (2). Cognitive impairment and OH are among the most frequent and troublesome non-motor symptoms in PD. In a community cohort of PD patients who survived 20 years from diagnosis, 83% had dementia and 48% had symptomatic OH (3, 4).

Orthostatic hypotension (OH) and cognitive dysfunction often coexist; however, whether the relationship is causative or associative is still unclear (5–8). Recently, there is growing evidence to suggest that several cognitive domains were reported to be associated with OH in PD, such as visuospatial, verbal memory, and attention (5, 9–13). Memory was the most consistent domain among the reports, and in particular, verbal memory was the only domain associated with OH in the patients before medication (14). However, these studies were based on limited data and were mainly cross-sectional, and these studies had often failed to adjust for important covariates such as age, disease severity, and disease duration.

Neuroimaging studies have provided considerable insight into the neurobiological basis of PD and its cognitive function (15). In a previous study, we investigated the impaired dynamic cerebral autoregulation (dCA) ability, which was obtained by transcranial doppler ultrasound (TCD) and beat-to-beat technology in PD patients with OH (16). Moreover, the impaired dCA is also associated with OH and Parkinson's disease dementia (PDD) (17). Meanwhile, there are some studies which indicated that PD patients with OH had a more severe impairment on tests of verbal immediate and delayed memory, and there were higher white matter lesion scores recorded by brain magnetic resonance imaging (MRI) in the OH group, which supported the vascular hypothesis (14, 18). Compared with dCA and MRI, 18fluorine-fluorodeoxyglucose positron emission tomography (18F-FDG-PET) provides more information on the underlying pathophysiology by highlighting the metabolic dysfunction of specific anatomical structures which can then be correlated with cognitive impairment (19). However, there are limited studies on the relationship between the cognitive impairment and PD with or without OH patients as shown by brain 18F-FDG PET patterns.

This cross-sectional cohort study aimed to investigate the characteristics of cognitive impairment with or without OH using the beat-to-beat and TCD technology by clinically defined PD and explore the associated dCA parameters, white matter hyperintensity (WMH) scores by brain MRI, and cerebral glucose metabolism with 18F-FDG-PET computed tomography (18F-FDG-PET/CT) among these patients. We aimed to (1) find out the relationship between the clinical or imaging indicators and PD-associated OH; (2) compare the characteristics of seven different cognitive domains (visuospatial/executive, naming, delayed recall verbal memory, attention, repetition/lexical fluency, similarities, and orientation) from the Montreal Cognitive Assessment (MoCA) Scale in PD patients with or without OH; and (3) figure out the correlation between imaging parameters and impaired cognitive domains of PD patients.

## 2. Methods and materials

### 2.1. Participants

Parkinson's disease patients (*n* = 31) were enrolled consecutively from the Department of Neurology of the Xuanwu Hospital, Capital Medical University in Beijing, China, from January 2021 to December 2021. All patients were diagnosed as under clinical diagnosis or probable diagnosis of PD according to the UK Brain Bank criteria, regardless of their disease severity. All diagnoses were established by two independent neurologists. The exclusion criteria were arterial hypertension, diabetes mellitus, arrhythmia, old myocardial infarction, cerebral vascular diseases, sleep disorders (i.e., rapid eye movement sleep behavior disorder, RBD), visual hallucinations and other mental diseases, dehydration, anemia or infection, severe systemic diseases, and poor cooperation. All the PD patients received dopaminergic therapy and without OH therapy. We evaluated the motor symptoms by Hoehn–Yahr (H–Y) stages and Movement Disorder Society Unified Parkinson's Disease Rating Scale (MDS-UPDRS) Part 3 scores. We evaluated all the PD patients for non-motor symptoms using the Non-Motor Symptom Scale (NMSS). We used MoCA to assess cognitive impairment [a total MoCA score cutoff < 26, which has 90% sensitivity and 75% specificity for PD mild cognitive impairment (MCI), a total MoCA score < 21, which has 81% sensitivity and 95% specificity for PD-dementia (20)] and the Hamilton Depression Scale (HAMD) to assess the severity of depression. All tests were performed in a quiet and temperature-controlled room to minimize stress and its effects on the patients.

### 2.2. Active standing test

Blood pressure (BP) measurements were performed in a silent, temperature-controlled room and the participants were asked to avoid alcohol, caffeine, nicotine, discontinue dopamine and vasoactive medications for 24 h before the examination. After 10 min of relaxation in the supine position, they were asked to take the active standing test, which involved lying in the supine position on the bed for 10 min and standing for 10 min (21). OH is defined as at least 20 mmHg drop in systolic BP and/or a 10 mmHg drop in diastolic BP within 3 min after standing (22). We distinguished neurogenic OH from non-neurogenic OH using the neurogenic OH ratio based on the active standing test (23), where the neurogenic OH was characterized by the Δheart rate/Δsystolic blood pressure (ΔHR/ΔSBP) ratio of < 0.492 during the active standing test (16). Based on these results, the participants were allocated either to the OH positive [OH(+)] group to the normal active standing test [OH(-)] group.

### 2.3. dCA measurement

Baseline brachial blood pressure was measured by a sphygmomanometer (Omron HBP-1300, Kyoto, Japan) in the supine position. During a 10-min supine period, three blood pressure readings were recorded. We used a servocontrolled plethysmograph (Finometer, Enschede, Netherlands) at middle finger to record the non-invasive continuous beat-to-beat BP (NIBP) and used a TCD (EMS-9D PRO, Shenzhen Delica Medical Equipment Co., Ltd., Shenzhen, China) to measure the cerebral blood flow velocity (CBFV) simultaneously in both the supine and standing positions during the entire procedure. Doppler probes were placed over the temporal window and fixed with an adjustable head frame. Continuous CBFV was measured in the left and right middle cerebral arteries (MCAs) at a depth of 50–65 mm (16, 17). All procedures were performed by a professional ultrasound doctor.

Based on the recommendations of the Cerebrovascular Research Network (CARNet) (24), the stored and processed data were adopted using the transfer function analysis (TFA) method to reflect the oscillations in BP and cerebral blood flow at a range of frequencies (17). With the assumption of linear correlation, it quantifies how much NIBP was reflected in the CBFV, and the regulator between NIBP and CBFV was indicated as cerebral autoregulation (16). The computer output parameters included the bilateral hemisphere phase, normalized gain (%/mmHg), absolute gain (cm/s/mmHg), and coherence at very low frequency (VLF, 0.02–0.07 Hz) and low frequency (LF, 0.07–0.2 Hz) separately. In general, the phase and gain reflect the temporal and amplitude relationship between BP and CBFV at the same frequency, whereas coherence approaches 1.0, reflected the linear relationship between oscillations in BP and CBFV (17). Thus, a lower phase and a higher gain represent a more impaired dCA ability. For the frequency domain, we evaluated within a VLF range, which was considered to reflect the most relevant real-time dynamic dCA behavior (25). We only estimated dCA parameters if the coherence ≥0.5 because a previous study had shown that a low coherence indicates that the linearity condition relating changes in velocity to pressure may be violated in this frequency range (26).

### 2.4. Brain MRI

A total of 31 patients underwent 3.0-T MRI (Magnetom Verio 3T; Siemens, Erlangen, Germany). Fluid-attenuated inversion recovery MRI sequences were reviewed for white matter changes and rated as per the modified Fazekas scale (27–29) as follows: grade 0, no white matter change; grade 1, minimal patchy white matter foci; grade 2, start of confluence of white matter disease; grade 3, large confluent areas; grade 4, confluence of white matter changes with cortical and subcortical involvement; grade 5, diffuse leukoencephalopathy with widespread and diffuse white matter disease.

### 2.5. PET/CT imaging

All PET/CT scans were performed on a uMI 510 PET/CT scanner (United Imaging Healthcare, Shanghai, China). 18F-FDG was produced in a radiochemistry laboratory, and the radiochemical purity of the tracer was >98%. After 40 min of intravenous injection of the radiotracer 18F-FDG (3.7 MBq/kg), 18F-FDG PET/CT brain scans were acquired. All patients were asked to fast for 4 to 6 h before injection. After injection, the patients were asked to have their eyes closed and keep quiet during the 40-min uptake period. The CT imaging thickness was 3.0 mm per slice and scanning time was 11.4 s. A single PET bed position was subsequently acquired for 15 min. Iterative reconstruction was performed with a 128 × 128 matrix and a 2.44-mm-thick slice. Two nuclear medicine physicians analyzed the 18F-FDG PET/CT images by visual assessment. The regions of interest (ROIs) are bilateral hippocampus and medial temporal lobes. Semiquantitative analysis was then completed by two experienced scientists (one from the Department of Nuclear Medicine and another from the Department of Radiology) who were double blinded to the results of the qualitative analysis. The ROI of each brain region was drawn along the outline of the gray matter cortex and mirrored to the contralateral side manually. The mean standard uptake value (SUV) of each ROI was quantitatively measured and recorded. To avoid individualized differences, the SUV of occipital lobe was treated as a reference, and we calculated the SUV (ROI) to SUV (occipital lobe) ratio.

### 2.6. Statistics

This research was designed as a single-center cross-sectional cohort study. Statistical analyses were performed using IBM SPSS v26 (IBM Corp., Armonk, NY, USA) and two-sided *p* < 0.05 were considered significant. Normally distributed continuous variables were expressed as the mean ± standard deviation (SD) and compared using *t*-tests, whereas skewed continuous variables were expressed as the median with interquartile range (IQR) and compared using the Mann–Whitney U test. Categorical variables, such as sex and cognitive function (i.e., the percentage of MCI/dementia), were expressed as percentages and analyzed using chi-square tests and a Fisher's exact test. Imaging parameters and the score of cognitive domains were tested with the Pearson correlation.

## 3. Results

### 3.1. Demographic information and cognition in PD patients with or without OH

Among our 31 PD patients (45.2% male), 20 (40% male) had OH and 11 (54.5% male) did not have OH. There were no significant differences (*p* > 0.05) between two groups in terms of sex, age, education years, disease duration, LED, H–Y stages, UPDRS Part 3 scores, NMSS scores, HAMD scores, MMSE scores, MoCA scores, and the percentage of MCI/dementia (Table 1).

**Table 1:** Demographic and clinical characteristics.

	**OH(+) (*n* = 20)**	**OH(-) (*n* = 11)**	***P***
Age, years	60.65 ± 8.18	58.64 ± 6.96	0.496
Sex, male (%)	8 (40.0%)	6 (54.5%)	0.532
Education, years	11.05 ± 3.50	10.73 ± 6.42	0.879
Disease duration, years	3.19 ± 3.17	3.55 ± 2.66	0.756
LED, mg	295.00 ± 299.26	256.80 ± 182.70	0.704
Hoehn and Yahr score	1.80 ± 0.73	2.18 ± 0.60	0.152
MDS-UPDRS Part 3 score	27.21 ± 15.45	30.40 ± 8.26	0.550
NMSS score	27.89 ± 30.20	27.40 ± 34.98	0.969
HAMD score	3.85 ± 3.18	6.45 ± 5.72	0.185
MMSE score	27.60 ± 2.91	27.91 ± 3.18	0.786
MoCA score	23.00 ± 5.04	23.55 ± 5.73	0.785
MCI (%)	2 (10.0%)	4 (36.4%)	0.151
Dementia (%)	8 (40.0%)	2 (18.2%)	0.202

OH, orthostatic hypotension; OH(+), OH positive; OH(-), OH negative; LED, levodopa equivalent dose; MDS-UPDRS, Movement Disorder Society Unified Parkinson's Disease Rating Scale; NMSS, Non-Motor Symptom Scale; HAMD, Hamilton Depression Scale; MMSE, Mini-mental State Examination; MoCA, Montreal Cognitive Assessment; MCI, mild cognitive impairment. Values are means (±SD), except sex (percentage of men), of the percentage of MCI and dementia (a total MoCA score cutoff < 26, which shows 90% sensitivity and 75% specificity for PD mild cognitive impairment (MCI) and a total MoCA score < 21, which shows 81% sensitivity and 95% specificity for PD-dementia).

### 3.2. Cognitive domains in the MoCA of PD patients with and without OH

**Figure 1:**
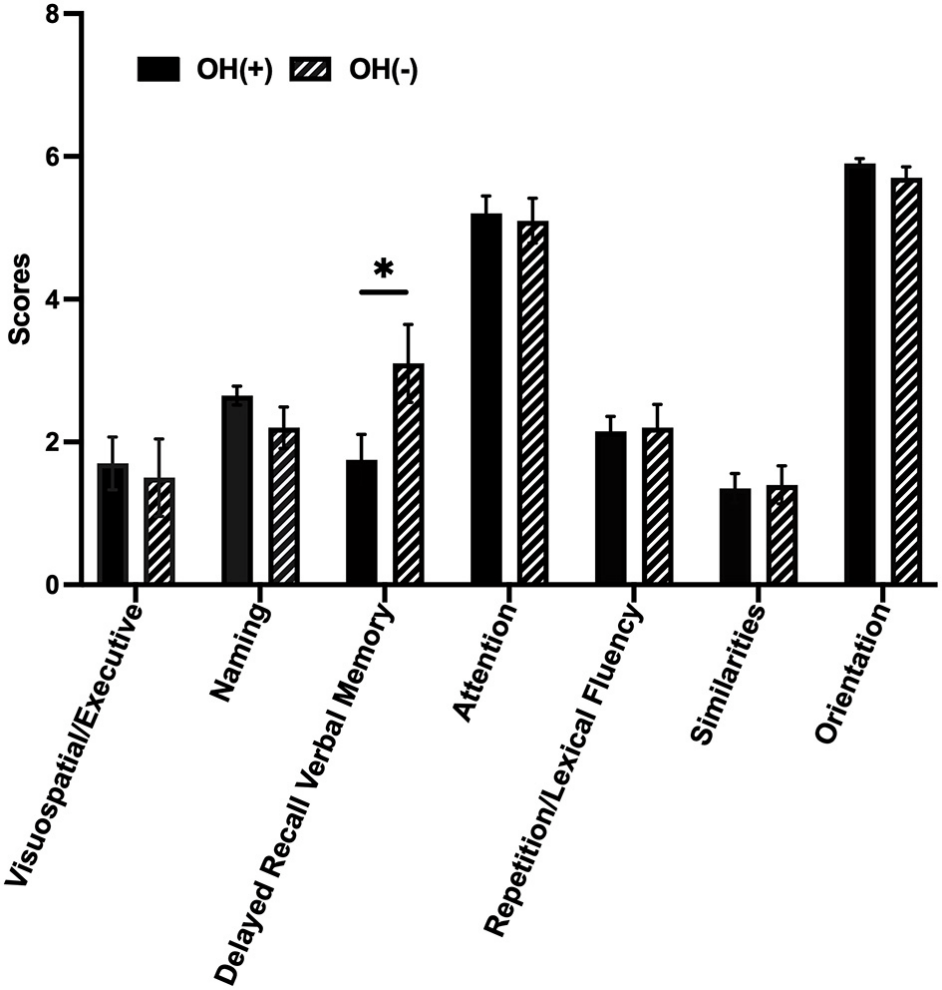
Cognitive domains of PD with or without OH. Column bars represent the scores of seven different cognitive domains by MoCA. The OH(+) group scored lower than the OH(-) group on delayed recall verbal memory function (1.75 ± 1.59 vs. 3.10 ± 1.73, *p* = 0.042, *p* < 0.05). OH, orthostatic hypotension; OH(+), OH positive; OH(-), OH negative. OH(+) (*n* = 20), OH(-) (*n* = 11). ^*^p < 0.05.

Among our 31 PD patients, the OH(+) group scored lower than the OH(-) group on delayed recall verbal memory function (1.75 ± 1.59 vs. 3.10 ± 1.73, *p* = 0.042); however, the scores on other six subdomains (visuospatial/executive, naming, attention, repetition/lexical fluency, similarities, and orientation) between two groups were not significantly different (Figure 1).

### 3.3. The characteristics of multiple imaging methods in PD patients with or without OH

**Figure 2:**
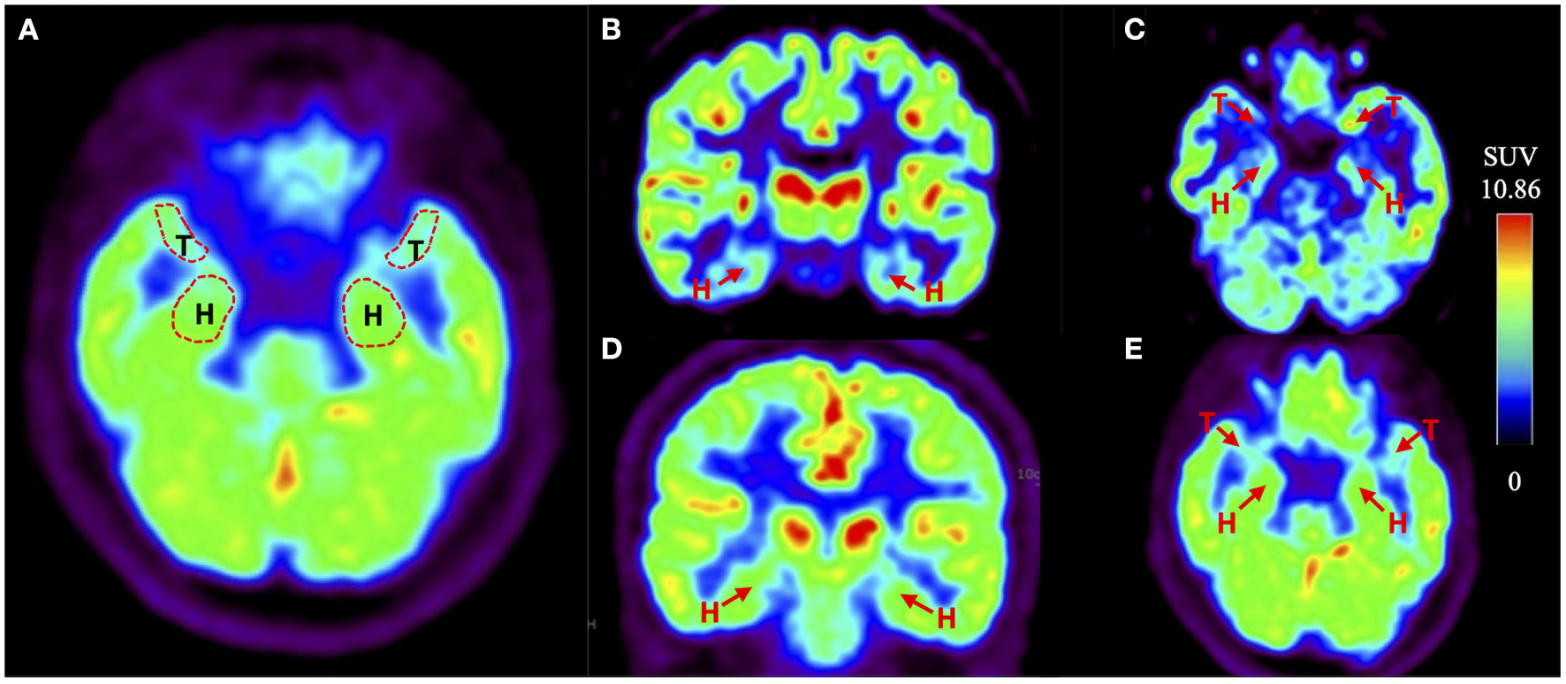
Example of the brain 18F-FDG PET/CT of PD with or without OH. **(A)** The red dotted line indicates the regions of interest (ROIs), which are bilateral hippocampus (H) and medial temporal lobes (T). **(B, C)** Shown are coronal and transverse sections of the brain 18F-FDG PET /CT of PD-OH(+) patients. The red arrow points the bilateral hippocampus (H) and medial temporal lobes (T). **(D, E)** Shown are coronal and transverse sections of the brain 18F-FDG PET/CT of PD-OH(-) patients. The red arrow points the bilateral hippocampus (H) and medial temporal lobes (T).

The data of imaging methods including dCA parameters (left and right VLF phase, left and right VLF gain) and the Fazekas scores by brain MRI and SUV (ROI) to SUV (occipital lobe) ratio calculated from the brain 18F-FDG PET/CT are presented in Table 2. The degree of bilateral VLF phase of patients with OH(+) was lower than that of OH(-) patients. At the same time, the bilateral VLF gain of patients with OH(+) was higher than that of OH(-) patients. Particularly, there was a significant difference in the right VLF gain between two groups (1.27 ± 0.17 vs. 1.10 ± 0.26, *p* = 0.045). The scores of WMH with OH(+) patients were higher than those of OH(-) PD patients, but there was no significant difference between two groups (*p* > 0.05). For the images (Figure 2) and parameters of the brain 18F-FDG PET/CT, the bilateral SUV (medial temporal lobe) to SUV (occipital lobe) ratio and the bilateral SUV (hippocampus) to SUV (occipital lobe) ratio were lower than those of OH(-) patients. Furthermore, there was a significant difference in the SUV (right medial temporal lobe) to SUV (occipital lobe) ratio (0.60 ± 0.08 vs. 0.67 ± 0.11, *p* = 0.049).

**Table 2:** Multiple imaging characteristics of PD with or without OH.

	**OH(+) (*n* = 20)**	**OH(-) (*n* = 11)**	***P***
**dCA parameters (MCA)**			
Left very low-frequency phase (degree)	56.47 ± 31.34	67.29 ± 9.05	0.120
Right very low-frequency phase (degree)	61.61 ± 21.07	64.82 ± 10.13	0.401
Left very low-frequency gain (%/mmHg)	1.30 ± 0.22	1.18 ± 0.20	0.417
Right very low-frequency gain (%/mmHg)	1.27 ± 0.17	1.10 ± 0.26	**0.045** ^*^
**Brain MRI**			
Fazekas score	0.77 ± 0.66	0.55 ± 0.69	0.368
**Brain 18F-FDG-PET/CT (ratio, SUV/SUV)**			
Left medial temporal lobe/occipital lobe	0.61 ± 0.09	0.64 ± 0.89	0.334
Right medial temporal lobe/occipital lobe	0.60 ± 0.08	0.67 ± 0.11	**0.049** ^*^
Left hippocampus/occipital lobe	0.79 ± 0.11	0.83 ± 0.10	0.261
Right hippocampus/occipital lobe	0.78 ± 0.16	0.84 ± 0.11	0.254

OH, orthostatic hypotension; OH(+), OH positive; OH(-), OH negative; dCA, dynamic cerebral autoregulation; MCA, middle cerebral artery; MRI, magnetic resonance imaging; 18F-FDG-PET/CT, 18fluorine-fluorodeoxyglucose positron emission tomography/computed tomography; SUV, standard uptake value. Values are means (±SD). ^*^p < 0.05. There was a significant difference in the right very low-frequency gain between OH(+) and OH(-) groups (1.27 ± 0.17 vs. 1.10 ± 0.26, p = 0.045) and there was a significant difference in the SUV (right medial temporal lobe) to SUV (occipital lobe) ratio (0.60 ± 0.08 vs. 0.67 ± 0.11, p = 0.049) between two groups.

### 3.4. The correlation between the delayed recall verbal memory function and imaging parameters in PD patients with or without OH

**Figure 3:**
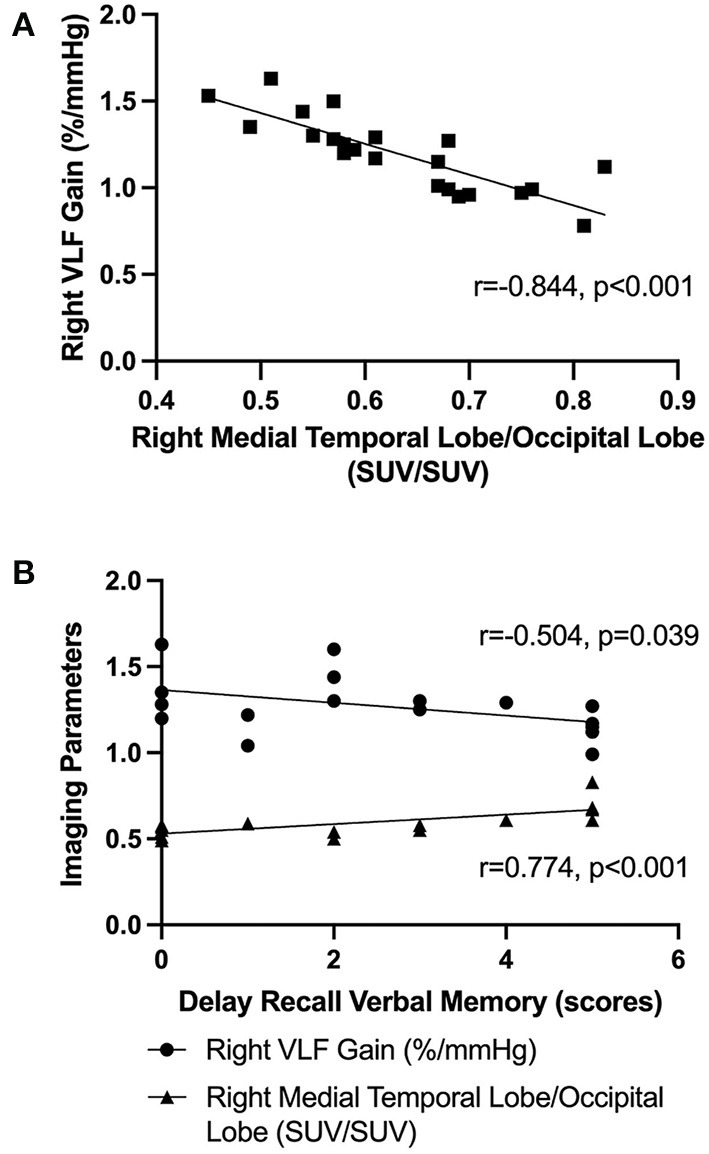
The Pearson correlation analysis. **(A)** The Pearson correlation analysis of the right VLF gain and the SUV (right medial temporal lobe) to SUV (occipital lobe) ratio in OH(+) and OH(-) groups. **(B)** The Pearson correlation analysis about the score of delay recall verbal memory function, the right VLF gain, and the SUV (right medial temporal lobe) to SUV (occipital lobe) ratio in OH(+) groups.

Our Pearson correlation analysis showed that the right VLF gain was negatively correlated with the SUV (right medial temporal lobe) to SUV (occipital lobe) ratio within the two groups (*r* = −0.844, *p* < 0.001) (Figure 3A). In the OH(+) group, the score of delayed recall verbal memory was positively correlated with the SUV (right medial temporal lobe) to SUV (occipital lobe) ratio (*r* = 0.774, *p* < 0.001), and the score of delayed recall verbal memory was negatively correlated with the right VLF gain (*r* = −0.504, *p* = 0.039) (Figure 3B), and such correlations were not found in the OH(-) group (*p* > 0.05).

## 4. Discussion

The major findings of this study are as follows: (1) delayed recall verbal memory deficits were the most prominent feature in OH(+) PD patients compared with OH(-) PD patients, (2) more impaired dCA ability and lower SUV (ROI) to SUV (occipital lobe) ratio by the brain 18F-FDG PET/CT were observed in PD patients with OH, especially the right VLF gain and the SUV (right medial temporal lobe) to SUV (occipital lobe) ratio, (3) the right VLF gain and the SUV (right medial temporal lobe) to SUV (occipital lobe) ratio were negatively correlated. In the OH(+) group, the score of delayed recall verbal memory was positively correlated with the SUV (right medial temporal lobe) to SUV (occipital lobe) ratio and was negatively correlated with the right VLF gain.

The delayed recall verbal memory function is thought to be dependent on the integrity of the Papez circuit, which includes the hippocampus, parahippocampal gyrus, medial temporal lobe, mammillary bodies, insula, and cingulate gyrus (30). Why was the delayed recall verbal memory impairment between OH(+) and OH(-) different? A possible mechanism for the contributory relationship was proposed to show that the intermittent cerebral hypoperfusion caused by episodic hypotension might induce brain key region ischemia (31–36). The cerebral hypoperfusion and anoxic damage in these vulnerable areas will lead to WMH and memory dysfunction (35, 36). Previous studies indicated that PD patients with OH had a more severe impairment on tests of verbal immediate and delayed memory, and there were higher WMH scores in the OH(+) group (14, 18). However, another study failed to find any difference in WMH between PD with and without OH (9). Our study showed that the score of WMH was higher in the OH(+) group than in the OH(-) group, but there was no significant difference between two groups (*p* > 0.05). The reason why the score of WMH did not show a significant difference within two groups might be attributed to the fact that the severity of WMH was rated using the semiquantitative visual rating system of Scheltens et al. by MRI (37), which is not the best approach to investigate the microstructure of deep brain white matter (30). The second reason may be that the severity of WMH could not represent the neuronal damage directly. Future studies should consider OH and WMH burdens in the pathological context of PD with more advanced neuroimaging approaches, such as diffusion kurtosis imaging (DKI) or diffusion tensor imaging (DTI). Although the role of WMH burdens in OH(+) PD patients is not clear, we still think that it could be a potential pathological mechanism for OH and cognitive impairment. As a result of WMH, PD patients may have had an early stage of cognitive impairment that was not detected yet.

As we all know, PD patients with OH appear to have poor cerebrovascular autoregulation (38). We attempted to use the dCA measurement to explain the reason why a delayed recall verbal memory impairment is more seriously experienced in OH(+) patients with PD. The dCA ability was obtained by the TCD and beat-to-beat technology. The TCD probes were placed over the temporal window and fixed with an adjustable head frame. The parameters of dCA represented the flow regulation function of the left and right MCAs that mainly supply blood to the temporal lobe and part of hippocampus (16). We evaluated the bilateral phase and gain within a VLF range, which was considered to reflect the most relevant real-time dynamic dCA behavior (25). Phase could be the representation of the time delay in the CBFV response to NIBP, where a higher phase means a better cerebral autoregulation ability. Gain represented the damping effect of dCA on the magnitude of BP oscillation. Absolute gain represents the absolute changes in NIBP and CBFV, whereas normalized gain represents relative changes in CBFV and NIBP, regardless of the baseline values of NIBP and CBFV. A higher gain represents a more impaired dCA ability. Our research showed there were more impaired dCA parameters in the OH(+) group, especially the right VLF gain, which indicated that CBFV was greatly influenced by NIBP on the right side. The result of cerebral autoregulation dysfunction might be attributed to the unstable flow through the distal capillary and injury to the cerebral microcirculation. This, in turn, will damage the microvascular system and induce several downstream sequelae, namely, the disruption of the blood–brain barrier, neuroinflammation, cerebral microbleeds, and white matter lesions (16). It is consistent with our hypothesis again that WMH burdens induced by dCA impairment might be the potential mechanism for memory dysfunction in PD with OH.

However, it is not enough to analyze the dCA ability alone, we also used the brain 18F-FDG PET/CT that focused on the regions of hippocampus and medial temporal lobe to clarify the relationship between the vascular mechanism and the memory impairment in OH(+) patients. As a diagnostic aid, 18F-FDG PET provides information that is currently considered as an additional feature for the diagnosis of PD (39). The previous 18F-FDG PET study showed that PD patients with visual hallucinations, indeed, show occipital hypometabolism and a high risk of developing dementia than patients without visual hallucinations (22, 23, 40). Likewise, PD patients with the RBD showed a lower cognitive performance and a higher likelihood of MCI and posterior cortical hypometabolism than PD patients without this disorder (39). The occipital lobe was chosen as the reference because we excluded diseases that might affect occipital lobe metabolism, for example RBD and visual hallucinations. Another reason was that we analyzed the metabolism of occipital lobe within two groups, and there was no significant difference between them (*p* > 0.05). Our research showed a lower SUV (right medial temporal lobe) to SUV (occipital lobe) ratio in the OH(+) group compared with the OH(-) group. The right VLF gain was negatively correlated with the SUV (right medial temporal lobe) to SUV (occipital lobe) ratio within two groups, which indicated the potential causal relationship between these two parameters. The brain 18F-FDG PET/CT provided an important information on the underlying pathophysiology that metabolic dysfunction of specific medial temporal lobe may be correlated with dCA dysfunction. Moreover, the score of delayed recall verbal memory was positively correlated with the SUV (right medial temporal lobe) to SUV (occipital lobe) ratio and was also negatively correlated with the right VLF gain in the OH(+) group, whereas such correlations were not found in the OH(-) group. The aforementioned conclusions suggested that the delayed recall verbal memory dysfunction in PD patients with OH might have been caused by the decreased metabolic dysfunction of specific medial temporal lobe due to the impaired dCA; however, it might not be the potential mechanism for the OH(-) group. Furthermore, we did not find any obvious metabolic dysfunction in the hippocampus region. The reason might be attributed to the fact that not only MCAs supply blood to the hippocampus, but also the posterior cerebral arteries (PCAs), and these dCA parameters cannot represent the autoregulation ability of PCAs. The reason why we only found significant differences in the imaging parameters on the right side is still not well explained. A previous study showed that pathological attenuation of neural activities within the right-lateralized cortical network is a neurophysiological biomarker of speech and limb movement timing deficits in PD (41). There is another study which found that memory-impaired PD patients demonstrated a more significant reduction in D2 receptor binding in the right medial temporal lobe compared to healthy controls and PD patients with no MCI (42). Future studies are needed to confirm whether PD with OH patients have right-lateralized reduction in D2 receptor binding, moreover, if there is any relationship between the memory impairment and speech or motor timing control in PD with OH.

Furthermore, animal models also suggested that cerebral hypoperfusion may decrease vascular clearance, increase cleavage of amyloid precursor protein, and promote beta amyloid accumulation (43). It is known that the beta amyloid protein is a well-recognized pathological biomarker for Alzheimer's disease (AD), whose typical symptoms are short-term memory deficits in the early stage (44, 45). Moreover, PD patients with coexisting AD pathology tend to have a higher level of α-synuclein accumulation in limbus and neocortex (46, 47). Additionally, hippocampus and medial temporal lobe are thought to play a prominent role in human episodic memory, and this pattern is often observed during verbal memory recall (48). Based on aforementioned studies and our conclusion, we suggest that another reason for an earlier delayed recall verbal memory dysfunction in OH with PD is that it is possible for OH to have overlapped pathological changes with AD, especially on certain areas related to the medial temporal–hippocampal circuit.

To the best of our knowledge, this is the first study to have elaborated on the relationship between the cognitive domain impairment of OH(+) and OH(-) in patients with PD using multiple neuroimaging parameters (dCA measurement, brain MRI, and 18F-FDG PET/CT). This study will provide new ideas for further research on the pathological mechanism of OH and cognitive impairment in PD. However, there are still limitations to this study. First, this study was based on limited data because we ruled out diseases that might affect the cerebral perfusion. Moreover, it was only a single-center cross-sectional study that cannot directly observe the progress of different cognitive domains in PD patients. Therefore, a larger longitudinal study is required to confirm our conclusion. Second, we did not distinguish the asymptomatic OH from symptomatic OH within the OH group, and healthy controls were not included in this study. Third, the assessment of MoCA is simple and feasible; however, it does not differentiate visuospatial function from executive function very well, and it also cannot well stratify with the memory dysfunction. More specific tests on memory function should be considered in future. Moreover, the other part of the Papez circuit of brain 18F-FDG PET/CT and AD-related biomarkers should be considered to further study the internal causes of impairment in different cognitive domains with OH in PD patients.

In conclusion, we found that PD with OH patients had poor delayed recall verbal memory function, which might have been caused by the decreased metabolic dysfunction of specific medial temporal lobe due to the impaired dCA of MCAs. An understanding of which of these mechanisms contributes to the link between the OH and cognitive function in PD may help clinicians to identify the most effective therapeutic strategy for OH patients with PD.

## Data availability statement

The original contributions presented in the study are included in the article/supplementary material, further inquiries can be directed to the corresponding authors.

## Ethics statement

The studies involving human participants were reviewed and approved by the Board of the Ethics Committee of Beijing Xuanwu Hospital, Capital Medical University. The patients/participants provided their written informed consent to participate in this study.

## Author contributions

XX and AH collected and conducted data analysis, interpreted the data, and drafted the manuscript for intellectual content. JZ and HS analyzed the data and interpreted the data. YX and PC revised the manuscript. LZ and EX acquired the study data, designed, and conceptualized the study. All authors contributed to the article and approved the submitted version.
